# A Crank–Nicolson finite spectral element method for the 2D non-stationary Stokes equations about vorticity–stream functions

**DOI:** 10.1186/s13660-018-1914-5

**Published:** 2018-11-19

**Authors:** Yanjie Zhou, Zhendong Luo, Fei Teng

**Affiliations:** 1 School of Science, Beijing Technology and Business University, Beijing, China; 2 School of Mathematics and Physics, North China Electric Power University, Beijing, China

**Keywords:** 65N30, 65N12, 65M15, Semi-discretized Crank–Nicolson format, The two-dimensional non-stationary Stokes equations about vorticity–stream functions, Fully discretized Crank–Nicolson finite spectral element format, Existence and stability as well as convergence

## Abstract

In this article, we first develop a semi-discretized Crank–Nicolson format about time for the two-dimensional non-stationary Stokes equations about vorticity–stream functions and analyze the existence, uniqueness, stability, and convergence of the semi-discretized Crank–Nicolson solutions. Then we establish a fully discretized Crank–Nicolson finite spectral element format based on the quadrilateral elements for the two-dimensional non-stationary Stokes equations about vorticity–stream functions and analyze the existence, uniqueness, stability, and convergence of the Crank–Nicolson finite spectral element solutions. In the end, we use three numerical examples to confirm the validity of our theoretical conclusions.

## Introduction

Let $\varTheta\subset \mathbb{R}^{2}$ be a connected and bounded domain. Consider the following two-dimensional (2D) non-stationary Stokes equations:

### Problem 1

Find $(u, v)$ and *p* such that

1 $$ \textstyle\begin{cases} \frac{\partial u}{\partial t}-\mu (\frac{\partial^{2} u}{\partial x^{2}}+ \frac{\partial^{2} u}{\partial y^{2}} ) +\frac{\partial p}{\partial x}= g_{1}, &(x,y,t)\in \varTheta\times(0,T), \\ \frac{\partial v}{\partial t}-\mu (\frac{\partial^{2} v}{\partial x^{2}}+ \frac{\partial^{2} v}{\partial y^{2}} ) +\frac{\partial p}{\partial y}= g_{2}, & (x,y,t)\in \varTheta\times(0,T), \\ \frac{\partial u}{\partial x}+ \frac{\partial v}{\partial y}=0, &(x,y,t)\in \varTheta\times(0,T), \\ u(x,y,t)= \varphi_{u}(x,y,t),\qquad v(x,y,t)= \varphi_{v}(x,y,t), &(x,y,t)\in\partial\varTheta\times (0,T], \\ u(x,y,0)= u^{0}(x,y),\qquad v(x,y,0)= v^{0}(x,y), &(x, y)\in \varTheta, \end{cases} $$

where $(u, v)$ represents the fluid velocity vector, *p* is the pressure, *T* is the total time, $\mu=1/\mathit{Re}$, *Re* is the Reynolds number, $g_{1}(x, y, t)$, $\varphi_{u}(x, y, t)$, and $u^{0}(x, y)$ are, respectively, the given body force, boundary value, and initial value functions in the *x* direction, and $g_{2}(x, y, t)$, $\varphi_{v}(x, y, t)$, $v^{0}(x,y)$ are, respectively, the given body force, boundary value, and initial value functions in the *y* direction.

For the sake of convenience but without losing generality, we will assume that $\varphi_{u}(x, y, t) =\varphi_{v}(x, y, t)=0$ in the following discussion.

The 2D non-stationary Stokes equations constitute an important mathematical model in fluid dynamics and have been successfully and extensively used to simulate the practical engineering problems as mentioned in [1–8]. However, when their computational domains are the irregular geometrical shape, we can usually not find their analytical solutions, so that we have to depend upon numerical solutions.

At present, finite difference (FD) scheme (see, e.g., [9, 10]), finite element (FE) method (see, e.g., [1–4, 11, 12]), finite volume element (FVE) method (see, e.g., [13, 14]), and spectral method (see [15–21]) are considered as to be four popular numerical methods. However, the spectral method holds highest accuracy among four numerical methods because it adopts the whole smooth functions (such as trigonometric functions, Chebyshev’s polynomials, Jacobi’s polynomials, and Legendre’s polynomials) to approximate unknown function, whereas the FE and FVE methods usually adopt standard polynomials to approximate unknown function and the FD scheme adopts difference quotient to approximate derivative. Especially, the finite spectral element (FSE) method can be suitable for the computational domains with complex geometric shapes, just as the FE method, so that it is widely used to solve various partial differential equations (PDEs), including the second-order elliptic equations, the parabolic equations, the hyperbolic equations, the hydromechanics equations (see, e.g., [22–26]).

Though some FSE methods have been presented in [25, 26], as far as we know, there has not been any report that the Crank–Nicolson (CN) finite spectral element (CNFSE) method is used to solve the 2D non-stationary Stokes equations about vorticity–stream functions, especially, there has not been any report about the theoretical analysis of the existence, stability, and convergence of the CNFSE solutions. Therefore, in this paper, we will first propose a time semi-discretized CN format with second-order time accuracy for the 2D non-stationary Stokes equations about vorticity–stream functions and analyze the errors of the time semi-discretized CN solutions. Then we will establish the fully discretized CNFSE format based on the quadrilateral elements for the 2D non-stationary Stokes equations about vorticity–stream functions and analyze the existence, uniqueness, stability, and convergence of the CNFSE solutions. In the end, we will use three numerical examples to confirm the validity of the obtained theoretical conclusions.

The CNFSE format for the 2D non-stationary Stokes equations about vorticity–stream functions has not only the second-order accuracy in time, but also is formed by system of two relatively independent linear equations for vorticity–stream approximate functions, so that it can easily be solved, which is different from the existing other FSE methods as mentioned above. Of course, the CNFSE format is also different from the spectral methods in [15–21]. Therefore, the CNFSE method here is a development and improvement over the existing methods.

The rest contents of this article is arranged as follows. In Sect. 2, we propose the semi-discretized CN format with approximation of second order by the time variable for the 2D non-stationary Stokes equations about vorticity–stream functions and analyze the existence, uniqueness, stability, and convergence of the time semi-discretized CN solutions. In Sect. 3, we establish the fully discretized CNFSE format based on the quadrilateral elements for the 2D non-stationary Stokes equations about vorticity–stream functions and analyze the existence, uniqueness, stability, and convergence of the CNFSE solutions. In Sect. 4, we use three numerical examples to confirm the validity of theoretical conclusion. Section 5 provides the main conclusions and discussions.

## The semi-discretized CN method about time for the 2D non-stationary Stokes equations

The Sobolev spaces, norms, and inner products used in this article are common (see [27]).

### The semi-discretized CN format about time

When *Θ* is connected and bounded and ${\partial u}/{\partial x}+{\partial v}/{\partial y}=0$, there is a unique stream function *ψ* such that

2 $$ u=\frac{\partial\psi}{\partial y},\qquad v=-\frac{\partial \psi}{\partial x}. $$

Further, there is unique a vorticity function *ω* such that $\omega={\partial v}/{\partial x}-{\partial u}/{\partial y}=-({\partial\psi^{2}}/{\partial x^{2}} +{\partial\psi^{2}}/{\partial y^{2}})$. Thus, Problem 1 can be transformed into the following system of two relatively independent linear PDEs about vorticity–stream functions.

#### Problem 2

Find *ω* and *ψ* such that

3 $$\begin{aligned}& \textstyle\begin{cases} -\frac{\partial\psi^{2}}{\partial x^{2}} -\frac{\partial\psi ^{2}}{\partial y^{2}}=\omega, &(x,y,t)\in\varTheta\times(0,T), \\ \psi=0,&(x,y,t)\in\partial\varTheta\times[0,T], \end{cases}\displaystyle \end{aligned}$$

4 $$\begin{aligned}& \textstyle\begin{cases} \frac{\partial\omega}{\partial t}-\mu (\frac{\partial ^{2}\omega}{\partial x^{2}} +\frac{\partial^{2}\omega}{\partial y^{2}} )=f, &(x,y,t)\in\varTheta\times(0,T), \\ \omega=0,&(x,y,t)\in\partial\varTheta\times[0,T], \\ \omega=\omega^{0},&{(x,y)}\in\varTheta, \end{cases}\displaystyle \end{aligned}$$

where $f={\partial g_{2}}/{\partial x}-{\partial g_{1}}/{\partial y}$, $\omega^{0}={\partial v^{0}}/{\partial x}-{\partial u^{0}}/{\partial y}$.

When $(g_{1}, g_{2})\in H^{1}(0,T; C^{1}(\bar{\varTheta}))\times H^{1}(0,T; C^{1}(\bar{\varTheta}))$ and $(u^{0}, v^{0})\in H^{2}(\varTheta)\times H^{2}(\varTheta )$, from the above discussion and the regularity for PDEs (see, e.g., [11, 27]) we can conclude that there is a unique solution $\omega\in H^{2}(0,T; C_{0}^{1}(\bar{\varTheta})\cap H^{2}(\varTheta))$ and $\psi \in H^{2}(0, T; C_{0}^{1}(\bar{\varTheta})\cap H^{2}(\varTheta))$ for Problem 2 meeting

5 $$ \|\psi\|_{H^{2}(W^{0,\infty})}+\|\psi\|_{H^{2}(H^{2})}+\|\omega\| _{H^{2}(W^{0,\infty})}+\|\omega\|_{H^{2}(H^{2})}\le\sigma\bigl(g_{1},g_{2}, u^{0}, v^{0},\mu\bigr), $$

where $\|\cdot\|_{H^{m}(W^{0,\infty})}$ and $\|\cdot\|_{H^{m}(H^{k})}$ represent, respectively, the norms in spaces $H^{m}(0, T; W^{0,\infty }(\varTheta))$ and $H^{m}(0, T; H^{k}(\varTheta))$, and $\sigma(g_{1},g_{2}, u^{0}, v^{0})$ is a non-negative constant dependent on $g_{1}$, $g_{2}$, $u^{0}$, $v^{0}$, and *μ*.

Let *M* be a positive integer, $\Delta t=T/M$ be the time step, and $\omega^{n}(x,y)$ and $\psi^{n}(x,y)$ be the approximations of $\omega (x,y,t)$ and $\psi(x,y, t)$ at $t_{n}=n\Delta t$ ($n=0, 1, 2, \ldots, M$), respectively. From the first equation in (4) we attain

6 $$ \frac{\partial^{2}\omega}{\partial t^{2}}=\frac{\partial}{\partial t} \biggl[\mu \biggl( \frac{\partial^{2}\omega}{\partial x^{2}}+\frac {\partial^{2}\omega}{\partial y^{2}} \biggr)+f \biggr]. $$

Thus, by Taylor’s formula and (6) we obtain

7 $$\begin{aligned} \frac{\partial\omega^{n-1}}{\partial t}&=\frac{\omega^{n}-\omega ^{n-1}}{\Delta t}-\frac{\Delta t}{2}\frac{\partial^{2}\omega ^{n-1}}{\partial t^{2}}+o \bigl(\Delta t^{2}\bigr) \\ &=\frac{\omega^{n}-\omega^{n-1}}{\Delta t}-\frac{\mu}{2} \biggl(\frac{\partial^{2}(\omega^{n}-\omega^{n-1})}{\partial x^{2}}+ \frac {\partial^{2}(\omega^{n}-\omega^{n-1})}{\partial y^{2}} \biggr) +\frac{ f^{n}-f^{n-1}}{2}{+o\bigl(\Delta t^{2} \bigr)}. \end{aligned}$$

Further, by inputting (7) into the first equation in (4) we obtain

8 $$ \frac{\omega^{n}-\omega^{n-1}}{\Delta t}-\frac{\mu}{2} \biggl[\frac {\partial^{2}(\omega^{n}+\omega^{n-1})}{\partial x^{2}}+ \frac{\partial ^{2}(\omega^{n}+\omega^{n-1})}{\partial y^{2}} \biggr] =\frac{ f^{n}+f^{n-1}}{2}{+o\bigl(\Delta t^{2} \bigr)}. $$

Set $V=H_{0}^{1}(\varTheta)$. Thus, by Green’s formula we can establish the semi-discretized CN format with the second-order accuracy in time as follows.

#### Problem 3

For given $\omega^{0}\in C^{0}(\bar{\varTheta})$ and $f^{n}\in C^{0}(\bar{\varTheta})$ ($n=0,1, \ldots, M$), find $(\omega^{n},\psi^{n})\in V\times V$ ($n=1, 2, \ldots, M$) that satisfy

9 $$\begin{aligned}& \int_{\varTheta}\biggl(\frac{\partial\psi^{n-1}}{\partial x}\frac{\partial w}{\partial x} + \frac{\partial\psi^{n-1}}{\partial y}\frac{\partial w}{\partial y} \biggr)\,\mathrm{d}x\,\mathrm{d}y \\& \quad = \int_{\varTheta}\omega^{n-1}w\,\mathrm{d}x\,\mathrm{d}y,\quad \forall w\in V, n=1, 2, \ldots, M+1; \end{aligned}$$

10 $$\begin{aligned}& \int_{\varTheta}\biggl[\omega^{n}w+\frac{\mu\Delta t}{2} \biggl(\frac{\partial\omega^{n}}{\partial x}\frac{\partial w}{\partial x} +\frac{\partial\omega^{n}}{\partial y}\frac{\partial w}{\partial y} \biggr) \biggr]\,\mathrm{d}x\,\mathrm{d}y \\& \quad = \int_{\varTheta}\biggl[\omega^{n-1}w-\frac{\mu\Delta t}{2} \biggl(\frac{\partial\omega^{n-1}}{\partial x}\frac{\partial w}{\partial x} +\frac{\partial\omega^{n-1}}{\partial y}\frac{\partial w}{\partial y} \biggr) \biggr]\,\mathrm{d}x\,\mathrm{d}y \\& \qquad {}+\frac{\Delta t}{2} \int_{\varTheta}\bigl(f^{n}+f^{n-1} \bigr)w\,\mathrm{d}x\,\mathrm{d}y,\quad \forall w\in V, n=1, 2, \ldots, M. \end{aligned}$$

### The existence, uniqueness, stability, and convergence of the time semi-discretized CN solutions

In the following, we employ the Lax–Milgram theorem, and the Hölder, Poincaré, Cauchy–Schwarz inequalities, and the following discrete Gronwall inequality (see [11, Lemma 3.4] or [28, Lemma 1.4.1]) to analyze the existence, uniqueness, stability, and convergence for the time semi-discretized CN solutions to Problem 3.

#### Lemma 4

*If* $\{a_{n}\}$ *and* $\{b_{n}\}$ *are two non*-*negative sequences*, *and* $\{c_{n}\}$ *is a positive monotone sequence*, *that satisfy*

$$ a_{n}+b_{n}\leq c_{n}+\bar{\lambda}\sum _{i=0}^{n-1}a_{i} \quad (\bar{\lambda }>0); \qquad a_{0}+b_{0}\leq c_{0}, $$

*then*

$$ a_{n}+b_{n}\leq c_{n}\exp(n\bar{\lambda}),\quad n=0, 1,2, \ldots. $$

We have the following main conclusion for Problem 3.

#### Theorem 5

*If* $\omega^{0}\in C^{0}(\bar{\varTheta})$ *and* $f^{n}\in C^{0}(\bar{\varTheta})$, *i*.*e*., $(u^{0},v^{0})\in C^{1}(\bar{\varTheta})\times C^{1}(\bar{\varTheta})$ *and* $(g_{1}^{n},g_{2}^{n})\in C^{1}(\bar{\varTheta})\times C^{1}(\bar{\varTheta})$, *the iterative equations* (9) *and* (10) *have a unique series of solutions* $(\omega^{n},\psi^{n})\in V\times V$ ($n=1, 2, \ldots, M$) *meeting the following stability*:

11 $$\begin{aligned}& \bigl\Vert \omega^{n} \bigr\Vert _{0}^{2}+{ \mu\Delta t} \bigl\Vert \nabla\omega^{n} \bigr\Vert _{0}^{2}\le \Biggl(2 \bigl\Vert \omega^{0} \bigr\Vert _{0}^{2}+\mu\Delta t \bigl\Vert \nabla \omega^{0} \bigr\Vert _{0}^{2}+{2\Delta t}\sum _{i=0}^{n} \bigl\Vert f^{i} \bigr\Vert _{0}^{2} \Biggr)\exp({2}n\Delta t), \\& \quad n=1, 2, \ldots, M; \end{aligned}$$

12 $$\begin{aligned}& \bigl\Vert \nabla\psi^{n}(x,y) \bigr\Vert _{0}\le\sigma \Biggl(2 \bigl\Vert \omega^{0} \bigr\Vert _{0}^{2}+ \mu \Delta t \bigl\Vert \nabla\omega^{0} \bigr\Vert _{0}^{2}+{2\Delta t}\sum_{i=0}^{n} \bigl\Vert f^{i} \bigr\Vert _{0}^{2} \Biggr)^{1/2}\exp(2n\Delta t), \\& \quad n=0, 1, 2, \ldots, M, \end{aligned}$$

*and the following convergence*:

13 $$ \bigl\Vert \psi(x,y,t_{n})-\psi^{n}(x,y) \bigr\Vert _{0,\infty}+ \bigl\Vert \omega(x,y,t_{n})-\omega ^{n}(x,y) \bigr\Vert _{0,\infty}\le\sigma\Delta t^{2}, $$

*where* *σ*, *used in the subsequent*, *is the generic positive constant independent of* Δ*t*, *but it is inequable in different places*.

#### Proof

Set

14 $$\begin{aligned}& \tilde{A}(\psi,w)= \int_{\varTheta}\biggl(\frac{\partial\psi}{\partial x}\frac{\partial w}{\partial x} + \frac{\partial\psi}{\partial y}\frac{\partial w}{\partial y} \biggr)\,\mathrm{d}x\,\mathrm{d}y,\quad \forall\psi, w\in V; \end{aligned}$$

15 $$\begin{aligned}& \tilde{G}(w)= \int_{\varTheta}\omega^{n-1}w\,\mathrm{d}x\,\mathrm{d}y,\quad \forall w\in V; \end{aligned}$$

16 $$\begin{aligned}& \tilde{B}(\omega,w)= \int_{\varTheta}\biggl[\omega w+\frac{\mu \Delta t}{2} \biggl( \frac{\partial\omega}{\partial x}\frac{\partial w}{\partial x} +\frac{\partial\omega}{\partial y}\frac{\partial w}{\partial y} \biggr) \biggr]\,\mathrm{d}x\,\mathrm{d}y,\quad \forall\omega, w\in V; \end{aligned}$$

17 $$\begin{aligned}& \begin{aligned}[b] \tilde{F}(w)&= \int_{\varTheta}\biggl[\omega^{n-1}w-\frac{\mu\Delta t}{2} \biggl(\frac{\partial\omega^{n-1}}{\partial x}\frac{\partial w}{\partial x} +\frac{\partial\omega^{n-1}}{\partial y}\frac{\partial w}{\partial y} \biggr) \biggr]\,\mathrm{d}x\,\mathrm{d}y\\ &\quad {}+\frac{\Delta t}{2} \int_{\varTheta}\bigl(f^{n}+f^{n-1} \bigr)w\,\mathrm{d}x\,\mathrm{d}y,\quad \forall w\in V. \end{aligned} \end{aligned}$$

Then Problem 3 can be rewritten as follows.

#### Problem 6

Find $(\omega^{n},\psi^{n})\in V\times V$ ($n=1, 2, \ldots, M$) that satisfy

18 $$\begin{aligned}& \tilde{A}\bigl(\psi^{n-1},w\bigr)=\tilde{G}(w),\quad \forall w\in V, n=1, 2, \ldots, M+1; \end{aligned}$$

19 $$\begin{aligned}& \tilde{B}\bigl(\omega^{n},w\bigr)=\tilde{F}(w),\quad \forall w\in V, n=1, 2, \ldots, M. \end{aligned}$$

Both bilinear functionals $\tilde{A}(\cdot,\cdot)$ and $\tilde {B}(\cdot,\cdot)$ are bounded and positive definitive on $V\times V$ and both linear functionals $\tilde{G}(\cdot)$ and $\tilde{F}(\cdot )$ are bounded on *V* for any given $\omega^{n-1}$, $f^{n}$, and $f^{n-1}$. Then, according on the Lax–Milgram theorem, Problem 6, i.e., the iterative equations (9) and (10), have a unique series of solutions $(\omega^{n},\psi^{n})\in V\times V$ ($n=1, 2, \ldots, M$).

By taking $w=\psi^{n-1}$ in (9) in addition to the Hölder and Poincaré inequalities we get

20 $$ \bigl\Vert \nabla\psi^{n-1} \bigr\Vert _{0}^{2}\le \bigl\Vert \omega^{n-1} \bigr\Vert _{0} \bigl\Vert \psi^{n-1} \bigr\Vert _{0}\le \sigma \bigl\Vert \omega^{n-1} \bigr\Vert _{0} \bigl\Vert \nabla\psi^{n-1} \bigr\Vert _{0},\quad n=1, 2, \ldots, M+1. $$

Further, we obtain

21 $$ \bigl\Vert \nabla\psi^{n-1} \bigr\Vert _{0}\le\sigma \bigl\Vert \omega^{n-1} \bigr\Vert _{0},\quad n=1, 2, \ldots, M+1. $$

By taking $w=\omega^{n}$ in (10) and the Hölder and Cauchy–Schwarz inequalities we obtain

22 $$\begin{aligned}& \bigl\Vert \omega^{n} \bigr\Vert _{0}^{2}+ \frac{\mu\Delta t}{2} \bigl\Vert \nabla\omega^{n} \bigr\Vert _{0}^{2} \\& \quad \le \bigl\Vert \omega^{n-1} \bigr\Vert _{0} \bigl\Vert \omega^{n} \bigr\Vert _{0}+\frac{\mu\Delta t}{2} \bigl\Vert \nabla\omega^{n-1} \bigr\Vert _{0} \bigl\Vert \nabla\omega^{n} \bigr\Vert _{0} \\& \qquad {}+{\frac{\Delta t}{2}} \bigl\Vert \omega^{n} \bigr\Vert _{0} \bigl\Vert f^{n} \bigr\Vert _{0}+{ \frac{\Delta t}{2}} \bigl\Vert \omega^{n} \bigr\Vert _{0} \bigl\Vert f^{n-1} \bigr\Vert _{0} \\& \quad \le \frac{1}{2} \bigl\Vert \omega^{n-1} \bigr\Vert _{0}^{2}+\frac{1}{2} \bigl\Vert \omega^{n} \bigr\Vert _{0}^{2}+\frac{\mu\Delta t}{4} \bigl\Vert \nabla\omega^{n-1} \bigr\Vert _{0}^{2}+ \frac{\mu \Delta t}{4} \bigl\Vert \nabla\omega^{n} \bigr\Vert _{0} \\& \qquad {}+{\frac{\Delta t}{2}} \bigl\Vert \omega^{n} \bigr\Vert _{0}^{2}+{\frac{\Delta t}{4}}\bigl( \bigl\Vert f^{n} \bigr\Vert _{0}^{2}+ \bigl\Vert f^{n-1} \bigr\Vert _{0}^{2}\bigr),\quad n=1, 2, \ldots, M. \end{aligned}$$

Further, we obtain

23 $$\begin{aligned}& \bigl\Vert \omega^{n} \bigr\Vert _{0}^{2}+ \frac{\mu\Delta t}{2} \bigl\Vert \nabla\omega^{n} \bigr\Vert _{0}^{2} \\& \quad \le \bigl\Vert \omega^{n-1} \bigr\Vert _{0}^{2}+ \frac{\mu\Delta t}{2} \bigl\Vert \nabla\omega ^{n-1} \bigr\Vert _{0}^{2} \\& \qquad {} +{\Delta t} \bigl\Vert \omega^{n} \bigr\Vert _{0}^{2}+{\frac{\Delta t}{2}}\bigl( \bigl\Vert f^{n} \bigr\Vert _{0}^{2}+ \bigl\Vert f^{n-1} \bigr\Vert _{0}^{2}\bigr),\quad n=1, 2, \ldots, M. \end{aligned}$$

Summing (23) from 1 to *n* yields

24 $$\begin{aligned}& \bigl\Vert \omega^{n} \bigr\Vert _{0}^{2}+ \frac{\mu\Delta t}{2} \bigl\Vert \nabla\omega^{n} \bigr\Vert _{0}^{2} \\& \quad \le \bigl\Vert \omega^{0} \bigr\Vert _{0}^{2}+ \frac{\mu\Delta t}{2} \bigl\Vert \nabla\omega^{0} \bigr\Vert _{0}^{2}+{\Delta t}\sum_{i=1}^{n} \bigl\Vert \omega^{i} \bigr\Vert _{0}^{2}+{ \Delta t}\sum_{i=0}^{n} \bigl\Vert f^{i} \bigr\Vert _{0}^{2},\quad n=1, 2, \ldots, M. \end{aligned}$$

When Δ*t* is sufficiently small such that $\Delta t\le1/2$, from (24), we attain

25 $$\begin{aligned}& \bigl\Vert \omega^{n} \bigr\Vert _{0}^{2}+{ \mu\Delta t} \bigl\Vert \nabla\omega^{n} \bigr\Vert _{0}^{2} \\& \quad \le2 \bigl\Vert \omega^{0} \bigr\Vert _{0}^{2}+ \mu\Delta t \bigl\Vert \nabla\omega^{0} \bigr\Vert _{0}^{2}+{2\Delta t}\sum_{i=0}^{n-1} \bigl\Vert \omega^{i} \bigr\Vert _{0}^{2}+{2{ \Delta t}}\sum_{i=0}^{n} \bigl\Vert f^{i} \bigr\Vert _{0}^{2}, \quad n=1, 2, \ldots, M. \end{aligned}$$

By using the discrete Gronwall inequality (Lemma 4) for (25), we obtain

26 $$\begin{aligned}& \bigl\Vert \omega^{n} \bigr\Vert _{0}^{2}+{ \mu\Delta t} \bigl\Vert \nabla\omega^{n} \bigr\Vert _{0}^{2}\le \Biggl(2 \bigl\Vert \omega^{0} \bigr\Vert _{0}^{2}+\mu\Delta t \bigl\Vert \nabla \omega^{0} \bigr\Vert _{0}^{2}+{2{\Delta t}}\sum _{i=0}^{n} \bigl\Vert f^{i} \bigr\Vert _{0}^{2} \Biggr)\exp({2}n\Delta t), \\& \quad n=1, 2, \ldots, M. \end{aligned}$$

This is exactly (11). By (11) and (21) we immediately attain (12).

By (8) we immediately attain (13).  □

## The CNFSE method for the 2D non-stationary Stokes equations about vorticity–stream functions

### The establishment of the CNFSE format

Let $\Im_{N}$ be the quasi-uniform quadrilateral subdivision on *Θ̄* and the spectral element subspace be chosen as the following:

27 $$ V_{N}=\bigl\{ w_{N}\in H^{1}_{0}( \varTheta)\cap C^{0}(\bar{\varTheta}): w_{N}|_{K_{j}}\in \mathcal{P}_{1}(K_{j}), K_{j}\in\Im_{N}, j=1,2, \ldots, N\bigr\} , $$

where *N* is the number of elements and $\mathcal{P}_{1}(K_{j})$ is formed by the quadrilateral spectral element, i.e.,

$$\mathcal{P}_{1}(K_{j})=\operatorname{span} \{N_{ij}: 1\le i\le4 \}, $$

the above $N_{ij}=\hat{N}_{i}\circ F_{j}^{-1}(x,y)$, $\hat{N}_{i}(\xi ,\eta)=[1+\cos\pi(\xi-\xi_{i})][1+\cos\pi(\eta-\eta_{i})]/4$, $(x,y)=F_{j}(\xi,\eta)= (\sum_{i=1}^{4}\hat{N}_{i}(\xi,\eta )x_{ij},\sum_{i=1}^{4}\hat{N}_{i}(\xi,\eta )y_{ij} )$ is a reversible transformation from $K_{j}\in\Im_{N}$ to the referencing quadrilateral $\hat{K}=[-1, 1]\times[-1, 1]$, and $(x_{ij}, y_{ij})$ and $(\xi_{i},\eta_{i})$ are the vertices of $K_{j}$ and *K̂*, respectively (see [11, 25]).

Let $R_{N}: H_{0}^{1}(\varTheta)\rightarrow V_{N}$ be the $H^{1}$-orthogonal projection, i.e., for any $\varphi\in H_{0}^{1}(\varTheta)$,

28 $$ \int_{\varTheta}\nabla(R_{N}\varphi- \varphi)\nabla v_{N}\,\mathrm{d}x\,\mathrm{d}y= 0,\quad \forall v_{N}\in V_{N}. $$

Further, because $\Im_{N}$ is the quasi-uniform quadrilateral subdivision for *Θ*, the number of nodes is approximately equal to the number of elements (see [11, Lemma 1.30]), $R_{N}$ has the following important property (see, e.g., [17, Chapters II and III]).

#### Theorem 7

*For any* $\varphi\in H^{q}(\varOmega)$ *with* $q\geq2$, *we have*

$$ \Vert \nabla R_{N}\varphi \Vert _{0,r}\le \sigma_{r} \Vert \nabla\varphi \Vert _{0,r},\qquad \bigl\Vert \partial^{k}(R_{N}\varphi-\varphi) \bigr\Vert _{0} \le\sigma N^{k-q}, \quad 0\leq k\leq q\leq N+1, $$

*where* $\sigma_{r}$ ($r=2$ *or* ∞, *and when* $r=2$, $\sigma_{r}=1$) *is the general positive constant independent of* *N* *and* *N* *is the number of nodes in* $\Im_{N}$.

By the subspace $V_{N}$ we can establish the CNFSE formulation as follows.

#### Problem 8

Find $(\omega_{N}^{n},\psi_{N}^{n})\in V_{N}\times V_{N}$ ($n=1, 2, \ldots, M$) that satisfy

29 $$\begin{aligned}& \int_{\varTheta}\biggl(\frac{\partial\psi_{N}^{n-1}}{\partial x}\frac{\partial w_{N}}{\partial x} + \frac{\partial\psi_{N}^{n-1}}{\partial y}\frac{\partial w_{N}}{\partial y} \biggr)\,\mathrm{d}x\,\mathrm{d}y \\& \quad = \int_{\varTheta}\omega_{N}^{n-1}w_{N}\,\mathrm{d}x\,\mathrm{d}y,\quad \forall w_{N}\in V_{N}, n=1, 2, \ldots, M+1; \end{aligned}$$

30 $$\begin{aligned}& \int_{\varTheta}\biggl[\omega_{N}^{n}w_{N}+ \frac{\mu\Delta t}{2} \biggl(\frac{\partial\omega_{N}^{n}}{\partial x}\frac{\partial w_{N}}{\partial x} +\frac{\partial\omega_{N}^{n}}{\partial y} \frac{\partial w_{N}}{\partial y} \biggr) \biggr]\,\mathrm{d}x\,\mathrm{d}y \\& \quad =\frac{\Delta t}{2} \int_{\varTheta}\bigl(f^{n}+f^{n-1} \bigr)w_{N}\,\mathrm{d}x\,\mathrm{d}y \\& \qquad {}+ \int_{\varTheta}\biggl[\omega_{N}^{n-1}w_{N}+ \frac{\mu\Delta t}{2} \biggl(\frac{\partial\omega_{N}^{n-1}}{\partial x}\frac{\partial w_{N}}{\partial x} +\frac{\partial\omega_{N}^{n-1}}{\partial y} \frac{\partial w_{N}}{\partial y} \biggr) \biggr]\,\mathrm{d}x\,\mathrm{d}y \\& \qquad {}-{\mu\Delta t} \int_{\varTheta}\biggl(\frac{\partial\omega _{N}^{n-1}}{\partial x}\frac{\partial w_{N}}{\partial x} + \frac{\partial\omega_{N}^{n-1}}{\partial y}\frac{\partial w_{N}}{\partial y} \biggr)\,\mathrm{d}x\,\mathrm{d}y, \\& \qquad \forall w_{N} \in V_{N}, n=1, 2, \ldots, M, \end{aligned}$$

where $\omega_{N}^{0}=R_{N}\omega^{0}$.

Set

$$\begin{aligned}& \boldsymbol{\omega}^{n}=\bigl(\omega_{11}^{n}, \omega_{21}^{n}, \omega_{31}^{n}, \omega_{41}^{n}, \omega_{12}^{n}, \omega_{22}^{n}, \omega_{32}^{n}, \omega _{42}^{n}, \ldots, \omega_{1N}^{n}, \omega_{2N}^{n},\omega_{3N}^{n}, \omega _{4N}^{n}\bigr)^{T}, \\& \boldsymbol{\psi}^{n}=\bigl(\psi_{11}^{n}, \psi_{21}^{n}, \psi_{31}^{n}, \psi _{41}^{n}, \psi_{12}^{n}, \psi_{22}^{n}, \psi_{32}^{n}, \psi_{42}^{n}, \ldots, \psi_{1N}^{n}, \psi_{2N}^{n}, \psi_{3N}^{n}, \psi_{4N}^{n}\bigr)^{T}, \\& \tilde{\boldsymbol {N}}=(N_{11}, N_{21}, N_{31}, N_{41}, N_{12}, N_{22}, N_{32}, N_{42}, \ldots, N_{1N},N_{2N}, N_{3N}, N_{4N})^{T}, \\& \omega _{N}^{n}=\sum_{j=1}^{N} \sum_{i=1}^{4}{\omega_{ij}^{n}}N_{ij}=: \tilde{\boldsymbol {N}}\cdot\boldsymbol{\omega}^{n}, \qquad \psi_{N}^{n}=\sum_{j=1}^{N} \sum_{i=1}^{4}{\psi _{ij}^{n}}N_{ij}=: \tilde {\boldsymbol {N}}\cdot\boldsymbol{\psi}^{n}. \end{aligned}$$

Thus, Problem 8 can be rewritten as follows.

#### Problem 9

Find $(\boldsymbol{\omega}^{n},\boldsymbol{\psi}^{n})\in \mathbb{R}^{4N}\times \mathbb{R}^{4N}$ ($n=1, 2, \ldots, M$) that satisfy

31 $$\begin{aligned}& \hat{\boldsymbol {A}}\boldsymbol{\psi}^{n-1}=\boldsymbol {C} \boldsymbol{\omega}^{n-1} ,\quad n=1, 2, \ldots, M+1; \end{aligned}$$

32 $$\begin{aligned}& \boldsymbol {A}\boldsymbol{\omega}^{n}=\boldsymbol {A} \boldsymbol{\omega}^{n-1}+\Delta t\boldsymbol {B}\boldsymbol{\omega}^{n-1}+\Delta t\boldsymbol {F}^{n},\quad n=1, 2, \ldots, M, \end{aligned}$$

where $\hat{\boldsymbol {A}}=\operatorname{diag}\{\hat{\boldsymbol {A}}_{11}, \hat{\boldsymbol {A}}_{22},\ldots,\hat{\boldsymbol {A}}_{NN} \}$, ${\boldsymbol {A}}=\operatorname{diag}\{{\boldsymbol {A}}_{11},{\boldsymbol {A}}_{22},\ldots,{\boldsymbol {A}}_{NN} \}$, ${\boldsymbol {B}}=\operatorname{diag}\{{\boldsymbol {B}}_{11},{\boldsymbol {B}}_{22},\ldots,{\boldsymbol {B}}_{NN} \}$, ${\boldsymbol {C}}=\operatorname{diag}\{{\boldsymbol {C}}_{11},{\boldsymbol {C}}_{22},\ldots,{\boldsymbol {C}}_{NN} \}$, $\boldsymbol {F}^{n}=(\boldsymbol {F}^{n}_{I\times1})_{4N\times1}$, and

$$\begin{aligned}& \hat{\boldsymbol {A}}_{IJ}= \biggl( \int_{\varTheta}\biggl[ \frac{\partial N_{iI}}{\partial x}\frac{\partial N_{jJ}}{\partial x}+ \frac{\partial N_{iI}}{\partial y}\frac{\partial N_{jJ}}{\partial y} \biggr]\,\mathrm{d}x\,\mathrm{d}y\biggr)_{4\times4}, \\& {\boldsymbol {A}}_{IJ}= \biggl( \int_{\varTheta}\biggl[ N_{iI}N_{jJ}+ \frac{\mu\Delta t}{2} \biggl(\frac{\partial N_{iI}}{\partial x}\frac{\partial N_{jJ}}{\partial x} +\frac{\partial N_{iI}}{\partial y} \frac{\partial N_{jJ}}{\partial y} \biggr) \biggr]\,\mathrm{d}x\,\mathrm{d}y\biggr)_{4\times4}, \\& {\boldsymbol {B}}_{IJ}=-{\mu} \biggl( \int_{\varTheta}\biggl(\frac{\partial N_{iI}}{\partial x}\frac{\partial N_{jJ}}{\partial x} + \frac{\partial N_{iI}}{\partial y}\frac{\partial N_{jJ}}{\partial y} \biggr) \,\mathrm{d}x\,\mathrm{d}y\biggr)_{4\times4}, \\& {\boldsymbol {C}}_{IJ}= \biggl( \int_{\varTheta}N_{iI}N_{jJ}\,\mathrm{d}x\,\mathrm{d}y\biggr)_{4\times4},\qquad \boldsymbol {F}^{n}_{I\times1}= \biggl( \int_{\varTheta}\frac {f^{(n)}+f^{(n-1)}}{2}N_{jI}\,\mathrm{d}x\,\mathrm{d}y\biggr)_{4\times1}. \end{aligned}$$

### The existence, stability, and convergence of the CNFSE solutions

To analyze the existence, stability, and convergence of the CNFSE solutions, we consider the max-norms of matrix and vector (the more detailed results see [26]), which are, respectively, defined dy

$$\begin{aligned}& \Vert {\boldsymbol {D}} \Vert _{\infty}=\max_{1\le i\le m}\sum _{j=1}^{l}|d_{ij}|,\quad \forall{ \boldsymbol {D}}=(d_{ij})_{m\times l}\in \mathbb{R}^{m}\times \mathbb{R}^{l}, \\& \Vert {\boldsymbol{\chi}} \Vert _{\infty}=\max_{1\le j\le m}| \chi_{j}|,\quad \forall{\boldsymbol {\chi}}=(\chi_{1}, \chi_{2}, \ldots, \chi_{m})^{T}\in \mathbb{R}^{m}. \end{aligned}$$

In the following, we employ the matrix theory, the FE (see [11]) and FSE (see [17]) methods, and the discrete Gronwall (Lemma 4), Hölder, Poincaré, and Cauchy–Schwarz inequalities to analyze the existence, stability, and convergence of the CNFSE solutions for Problem 8. We have the following result.

#### Theorem 10

*If* $\omega^{0}={\partial v^{0}}/{\partial x}-{\partial u^{0}}/{\partial y}\in W^{0,\infty}(\varTheta)$, *i*.*e*., $(u^{0},v^{0})\in W^{1,\infty}(\varTheta )\times W^{1,\infty}(\varTheta)$, *and* $f={\partial g_{2}}/{\partial x}-{\partial g_{1}}/{\partial y}\in W^{0,\infty}(\varTheta)$, *i*.*e*., $(g_{1},g_{2})\in W^{1,\infty}(\varTheta)\times W^{1,\infty}(\varTheta)$, *then the CNFSE solutions* $(\omega_{N}^{n}, \psi_{N}^{n})$ *are existing and unique and satisfy the following stability*:

33 $$\begin{aligned}& \bigl\Vert \omega_{N}^{n} \bigr\Vert _{0,\infty}\le\sigma \Biggl( \bigl\Vert \boldsymbol{\omega}^{0} \bigr\Vert _{\infty}+\Delta t N^{-1}\sum _{i=1}^{n} \bigl\Vert \boldsymbol {F}^{i} \bigr\Vert _{\infty } \Biggr),\quad n=1, 2, \ldots, M, \end{aligned}$$

34 $$\begin{aligned}& \bigl\Vert \psi_{N}^{n} \bigr\Vert _{0,\infty}\le\sigma N^{-1} \Biggl( \bigl\Vert \boldsymbol {\omega}^{0} \bigr\Vert _{\infty}+\Delta t N^{-1}\sum _{i=1}^{n} \bigl\Vert \boldsymbol {F}^{i} \bigr\Vert _{\infty} \Biggr),\quad n=1, 2, \ldots, M. \end{aligned}$$

*where* *σ*, *used in the subsequent*, *is also the generic positive constant independent of* Δ*t* *and* *N*, *but is inequable in different places*. *Further*, *when* $(\omega,\psi)\in [H^{3}(0,T;H^{q}(\varTheta)\cap H_{0}^{1}(\varTheta))]\times [H^{3}(0,T;H^{q}(\varTheta)\cap H_{0}^{1}(\varTheta))]$ ($2\le q\le N+1$), *we have the following error estimates*:

35 $$\begin{aligned}& \bigl\Vert \omega(x, y, t_{n})-\omega_{N}^{n} \bigr\Vert _{0}+\sqrt{\Delta t} \bigl\Vert \nabla\bigl(\omega(x, y, t_{n})-\omega_{N}^{n}\bigr) \bigr\Vert _{0}\le\sigma \bigl(\Delta t^{2}+N^{1-q} \bigr); \end{aligned}$$

36 $$\begin{aligned}& \bigl\Vert \nabla\bigl(\psi(x, y, t_{n})- \psi_{N}^{n}\bigr) \bigr\Vert _{0} \le\sigma \bigl(\Delta t^{2}+N^{1-q} \bigr), \end{aligned}$$

*where* $n=1, 2, \ldots, M$.

#### Proof

First, by the symmetry and positive definiteness of the matrices $\hat{\boldsymbol {A}}$ and ***A*** we conclude that Problem 9 has a unique series of the coefficient vector solutions $(\boldsymbol{\omega}^{n},\boldsymbol{\psi}^{n})\in \mathbb{R}^{4N}\times \mathbb{R}^{4N}$ ($n=1, 2, \ldots, M$). Thus, by $\omega_{N}^{n}=\tilde{\boldsymbol {N}}\cdot\boldsymbol{\omega}^{n}$, $\psi_{N}^{n}=\tilde{\boldsymbol {N}}\cdot\boldsymbol{\psi}^{n}$ we can immediately conclude that Problem 8 has a unique series of the CNFSE solutions $(\omega_{N}^{n}, \psi_{N}^{n})$ ($n=1, 2, \ldots, M$).

Next, we analyze the stability of the CNFSE solutions. From (31) and (32) we can attain the following:

37 $$ \textstyle\begin{cases} \boldsymbol{\psi}^{n-1}={\hat{\boldsymbol {A}}^{-1}}\boldsymbol {C}\boldsymbol{\omega}^{n-1},& 1\leq n\leq M+1; \\ \boldsymbol{\omega}^{n}=\boldsymbol{\omega}^{n-1}+\Delta t{\boldsymbol {A}}^{-1}\boldsymbol {B}\boldsymbol{\omega}^{n-1}+\Delta t{\boldsymbol {A}}^{-1}\boldsymbol {F}^{n},& 1\leq n \leq M. \end{cases} $$

Moreover, from the FE method (see, e.g., [11, Lemmas 1.18 and 1.22]) and FSE method (see, e.g., [17, Chapters II and III]) we can attain the following inequalities:

38 $$ \begin{aligned} & \bigl\Vert \hat{\boldsymbol {A}}^{-1} \bigr\Vert _{\infty}\le\sigma N^{-1};\qquad \bigl\Vert { \boldsymbol {A}}^{-1} \bigr\Vert _{\infty}\le\sigma N^{-1}; \qquad \Vert {\boldsymbol {B}} \Vert _{\infty}\le\sigma N; \\ & \Vert {\boldsymbol {C}} \Vert _{\infty}\le\sigma,\qquad \bigl\Vert { \boldsymbol {C}}^{-1} \bigr\Vert _{\infty}\le\sigma. \end{aligned} $$

Thus, by (37) and (38) we obtain

39 $$\begin{aligned}& \bigl\Vert \boldsymbol{\psi}^{n} \bigr\Vert _{\infty}\le\sigma N^{-1} \bigl\Vert \boldsymbol {\omega}^{n} \bigr\Vert _{\infty},\quad n=0, 1, 2, \ldots, M; \end{aligned}$$

40 $$\begin{aligned}& \bigl\Vert \boldsymbol{\omega}^{n} \bigr\Vert _{\infty}\le \bigl\Vert \boldsymbol{\omega}^{n-1} \bigr\Vert _{\infty }+ \sigma\Delta t \bigl\Vert \boldsymbol{\omega}^{n-1} \bigr\Vert _{\infty}+\sigma\Delta t N^{-1} \bigl\Vert \boldsymbol {F}^{n} \bigr\Vert _{\infty},\quad n=1, 2, \ldots, M. \end{aligned}$$

Summing (40) from 1 to *n*, we attain

41 $$ \bigl\Vert \boldsymbol{\omega}^{n} \bigr\Vert _{\infty}\le \bigl\Vert \boldsymbol{\omega}^{0} \bigr\Vert _{\infty}+ \sigma \Delta t\sum_{i=0}^{n-1} \bigl\Vert \boldsymbol{\omega}^{i} \bigr\Vert _{\infty}+\sigma \Delta t N^{-1}\sum_{i=1}^{n} \bigl\Vert \boldsymbol {F}^{i} \bigr\Vert _{\infty},\quad n=1, 2, \ldots, M. $$

By the discrete Gronwall inequality (Lemma 4) and from (41) we obtain

42 $$ \bigl\Vert \boldsymbol{\omega}^{n} \bigr\Vert _{\infty}\le \Biggl( \bigl\Vert \boldsymbol{\omega}^{0} \bigr\Vert _{\infty }+\sigma\Delta t N^{-1}\sum _{i=1}^{n} \bigl\Vert {\boldsymbol {F}^{i}} \bigr\Vert _{\infty} \Biggr)\exp[\sigma n\Delta t], \quad n=1, 2, \ldots, M. $$

Combining (42) with (39), we get

43 $$ \bigl\Vert \boldsymbol{\psi}^{n} \bigr\Vert _{\infty}\le\sigma N^{-1} \Biggl( \bigl\Vert \boldsymbol {\omega}^{0} \bigr\Vert _{\infty}+\Delta t N^{-1}\sum _{i=1}^{n} \bigl\Vert \boldsymbol {F}^{i} \bigr\Vert _{\infty} \Biggr), \quad n=1, 2, \ldots, M. $$

Because $\omega_{N}^{n}=\tilde{\boldsymbol {N}}\cdot\boldsymbol{\omega}^{n}$, $\psi _{N}^{n}=\tilde{\boldsymbol {N}}\cdot\boldsymbol{\psi}^{n}$, and $\|\tilde{\boldsymbol {N}}\| _{\infty}\le1$, from (42) and (43) we immediately attain (33) and (34), respectively.

Finally, we discuss the convergence of the CNFSE solutions. Subtracting (29) and (30) from (9) and (10) taking $w=w_{N}$, respectively, we attain the following equations for determining the error:

44 $$\begin{aligned}& \int_{\varTheta}\nabla\bigl(\psi^{n-1}-\psi_{N}^{n-1} \bigr)\nabla w_{N}\,\mathrm{d}x\,\mathrm{d}y \\& \quad = \int_{\varTheta}\bigl(\omega^{n-1}-\omega_{N}^{n-1} \bigr)w_{N}\,\mathrm{d}x\,\mathrm{d}y,\quad \forall w_{N}\in V_{N}, n=1, 2, \ldots, M+1; \end{aligned}$$

45 $$\begin{aligned}& \int_{\varTheta}\biggl[\bigl(\omega^{n}- \omega_{N}^{n}\bigr)w_{N}+\frac{\mu \Delta t}{2}\nabla \bigl(\omega^{n}-\omega_{N}^{n}\bigr)\nabla w_{N} \biggr]\,\mathrm{d}x\,\mathrm{d}y \\& \quad = \int_{\varTheta}\bigl(\omega^{n-1}-\omega_{N}^{n-1} \bigr)w_{N}\,\mathrm{d}x\,\mathrm{d}y \\& \qquad {}-\frac{\mu\Delta t}{2} \int_{\varTheta}\nabla\bigl(\omega ^{n-1}- \omega_{N}^{n-1}\bigr)\nabla w_{N}\,\mathrm{d}x\,\mathrm{d}y,\quad \forall w_{N}\in V_{N}, n=1, 2, \ldots, M, \end{aligned}$$

where $\omega_{N}^{0}=R_{N}\omega^{0}$.

By (44) and (28), the Cauchy–Schwarz, Hölder, and Poincaré inequalities, and Theorem 7, we obtain

46 $$\begin{aligned}& \bigl\Vert \nabla\bigl(\psi^{n-1}-\psi_{N}^{n-1} \bigr) \bigr\Vert _{0}^{2} \\& \quad = \int_{\varTheta}\nabla \bigl(\psi^{n-1}-\psi_{N}^{n-1} \bigr)\nabla\bigl(\psi^{n-1}-\psi_{N}^{n-1}\bigr)\,\mathrm{d}x\,\mathrm{d}y \\& \quad = \int_{\varTheta}\nabla\bigl(\psi^{n-1}-R_{N} \psi^{n-1}\bigr)\nabla\bigl(\psi ^{n-1}-R_{N} \psi^{n-1}\bigr)\,\mathrm{d}x\,\mathrm{d}y \\& \qquad {}+ \int_{\varTheta}\nabla\bigl(\psi^{n-1}-\psi_{N}^{n-1} \bigr)\nabla\bigl(R_{N}\psi ^{n-1}-\psi_{N}^{n-1} \bigr)\,\mathrm{d}x\,\mathrm{d}y \\& \quad = \bigl\Vert \nabla\bigl(\psi^{n-1}-R_{N} \psi^{n-1}\bigr) \bigr\Vert _{0}^{2}+ \int_{\varTheta}\bigl(\omega ^{n-1}-\omega_{N}^{n-1} \bigr) \bigl(R_{N}\psi^{n-1}-\psi_{N}^{n-1} \bigr)\,\mathrm{d}x\,\mathrm{d}y \\& \quad \le \bigl\Vert \nabla\bigl(\psi^{n-1}-R_{N} \psi^{n-1}\bigr) \bigr\Vert _{0}^{2}+ \bigl\Vert \omega^{n-1}-\omega _{N}^{n-1} \bigr\Vert _{0} \bigl\Vert R_{N}\psi^{n-1}- \psi^{n-1} \bigr\Vert _{0} \\& \qquad {}+ \bigl\Vert \omega^{n-1}-\omega_{N}^{n-1} \bigr\Vert _{0} \bigl\Vert \psi^{n-1}- \psi_{N}^{n-1} \bigr\Vert _{0} \\& \quad \le\sigma\bigl(N^{2-2q}+ \bigl\Vert \omega^{n-1}- \omega_{N}^{n-1} \bigr\Vert _{0}^{2} \bigr) \\& \qquad {}+\frac{1}{2} \bigl\Vert \nabla\bigl(\psi^{n-1}- \psi_{N}^{n-1}\bigr) \bigr\Vert _{0}^{2}, \quad n=1, 2, \ldots, M+1, 2\le q\le N+1. \end{aligned}$$

Further, we get

47 $$\begin{aligned}& \bigl\Vert \nabla\bigl(\psi^{n-1}-\psi_{N}^{n-1} \bigr) \bigr\Vert _{0} \\& \quad \le\sigma\bigl(N^{1-q}+ \bigl\Vert \omega^{n-1}- \omega_{N}^{n-1} \bigr\Vert _{0}\bigr), \quad n=1, 2, \ldots, M+1, 2\le q\le N+1. \end{aligned}$$

By using (45) and (28), the Hölder, Poincaré, and Cauchy–Schwarz inequalities, and Theorem 7, we obtain

48 $$\begin{aligned}& \bigl\Vert \omega^{n}-\omega_{N}^{n} \bigr\Vert _{0}^{2}+\frac{\mu\Delta t}{2} \bigl\Vert \nabla\bigl( \omega^{n}-\omega_{N}^{n}\bigr) \bigr\Vert _{0}^{2} \\& \quad = \int_{\varTheta}\bigl(\omega^{n}-\omega_{N}^{n} \bigr) \bigl(\omega^{n}-\omega _{N}^{n}\bigr)\,\mathrm{d}x\,\mathrm{d}y+\frac{\mu\Delta t}{2} \int_{\varTheta}\nabla\bigl(\omega ^{n}- \omega_{N}^{n}\bigr)\nabla\bigl(\omega^{n}- \omega_{N}^{n}\bigr)\,\mathrm{d}x\,\mathrm{d}y \\& \quad = \int_{\varTheta}\bigl(\omega^{n}-\omega_{N}^{n} \bigr) \bigl(\omega^{n}-R_{N}\omega ^{n}\bigr)\,\mathrm{d}x\,\mathrm{d}y+\frac{\mu\Delta t}{2} \bigl\Vert \nabla\bigl(\omega^{n}-R_{N} \omega ^{n}\bigr) \bigr\Vert _{0}^{2} \\& \qquad {}+ \int_{\varTheta}\bigl(\omega^{n}-\omega_{N}^{n} \bigr) \bigl(R_{N}\omega^{n}-\omega _{N}^{n} \bigr)\,\mathrm{d}x\,\mathrm{d}y \\& \qquad {}+\frac{\mu\Delta t}{2} \int_{\varTheta}\nabla\bigl(\omega ^{n}- \omega_{N}^{n}\bigr)\nabla\bigl(R_{N} \omega^{n}-\omega_{N}^{n}\bigr)\,\mathrm{d}x\,\mathrm{d}y \\& \quad = \int_{\varTheta}\bigl(\omega^{n}-\omega_{N}^{n} \bigr) \bigl(\omega^{n}-R_{N}\omega ^{n}\bigr)\,\mathrm{d}x\,\mathrm{d}y+\frac{\mu\Delta t}{2} \bigl\Vert \nabla\bigl(\omega^{n}-R_{N} \omega ^{n}\bigr) \bigr\Vert _{0}^{2} \\& \qquad {}+ \int_{\varTheta}\bigl(\omega^{n-1}-\omega_{N}^{n-1} \bigr) \bigl(R_{N}\omega^{n}-\omega ^{n}\bigr)\,\mathrm{d}x\,\mathrm{d}y+ \int_{\varTheta}\bigl(\omega^{n-1}-\omega_{N}^{n-1} \bigr) \bigl(\omega ^{n}-\omega_{N}^{n}\bigr)\,\mathrm{d}x\,\mathrm{d}y \\& \qquad {}-\frac{\mu\Delta t}{2} \int_{\varTheta}\nabla\bigl(\omega ^{n-1}-R_{N} \omega^{n-1}\bigr)\nabla\bigl(R_{N}\omega^{n}- \omega^{n}\bigr)\,\mathrm{d}x\,\mathrm{d}y \\& \qquad {}-\frac{\mu\Delta t}{2} \int_{\varTheta}\nabla\bigl(\omega^{n-1}-\omega _{N}^{n-1}\bigr)\nabla\bigl(\omega^{n}- \omega_{N}^{n}\bigr)\,\mathrm{d}x\,\mathrm{d}y \\& \quad \le\sigma N^{-q}\bigl( \bigl\Vert \omega^{n}- \omega_{N}^{n} \bigr\Vert _{0}+ \bigl\Vert \omega ^{n-1}-\omega_{N}^{n-1} \bigr\Vert _{0}\bigr)+\sigma\Delta tN^{2-2q} \\& \qquad {}+\frac{\mu\Delta t}{4} \bigl\Vert \nabla\bigl(\omega^{n-1}-R_{N} \omega^{n-1}\bigr) \bigr\Vert _{0}^{2}+ \frac{\mu\Delta t}{4} \bigl\Vert \nabla\bigl(\omega^{n}-R_{N} \omega^{n}\bigr) \bigr\Vert _{0}^{2} \\& \qquad {}+\frac{1}{2} \bigl\Vert \omega^{n-1}- \omega_{N}^{n-1} \bigr\Vert _{0}^{2}+ \frac{1}{2} \bigl\Vert \omega ^{n}-\omega_{N}^{n} \bigr\Vert _{0}^{2},\quad n=1, 2, \ldots, M, 2\le q\le N+1. \end{aligned}$$

Further, we get

49 $$\begin{aligned}& \bigl\Vert \omega^{n}-\omega_{N}^{n} \bigr\Vert _{0}^{2}+\frac{\mu\Delta t}{2} \bigl\Vert \nabla\bigl( \omega^{n}-\omega_{N}^{n}\bigr) \bigr\Vert _{0}^{2} \\& \quad \le \bigl\Vert \omega^{n-1}-\omega _{N}^{n-1} \bigr\Vert _{0}^{2}+\frac{\mu\Delta t}{2} \bigl\Vert \nabla \bigl(\omega^{n-1}-\omega _{N}^{n-1}\bigr) \bigr\Vert _{0}^{2} \\& \qquad {} +\sigma N^{-q}\bigl( \bigl\Vert \omega^{n}- \omega_{N}^{n} \bigr\Vert _{0}+ \bigl\Vert \omega ^{n-1}-\omega_{N}^{n-1} \bigr\Vert _{0}\bigr)+\sigma\Delta tN^{2-2q},\quad n=1, 2, \ldots, M. \end{aligned}$$

Summing (49) from 1 to *n* and using Theorem 7, we attain

50 $$\begin{aligned}& \bigl\Vert \omega^{n}-\omega_{N}^{n} \bigr\Vert _{0}^{2}+\frac{\mu\Delta t}{2} \bigl\Vert \nabla\bigl( \omega^{n}-\omega_{N}^{n}\bigr) \bigr\Vert _{0}^{2} \\& \quad \le \bigl\Vert \omega^{0}-R_{N}\omega ^{0} \bigr\Vert _{0}^{2}+\frac{\mu\Delta t}{2} \bigl\Vert \nabla\bigl(\omega^{0}-R_{N}\omega ^{0}\bigr) \bigr\Vert _{0}^{2} \\& \qquad {}+\sigma N^{-1}\sum_{i=0}^{n} \bigl\Vert \omega^{i}-\omega_{N}^{i} \bigr\Vert _{0}^{2}+\sigma \bigl(N^{2-2q}+n\Delta tN^{2-2q}\bigr) \\& \quad \le\sigma\bigl(N^{2-2q}+n\Delta tN^{2-2q}\bigr)+ \frac{1}{2N}\sum_{i=0}^{n} \bigl\Vert \omega^{i}-\omega_{N}^{i} \bigr\Vert _{0}^{2}, \quad n=1, 2, \ldots, M. \end{aligned}$$

When *N* is sufficiently large such that $N^{-1}\le1/2$, from (50) we attain

51 $$\begin{aligned}& \bigl\Vert \omega^{n}-\omega_{N}^{n} \bigr\Vert _{0}^{2}+\Delta t \bigl\Vert \nabla\bigl(\omega ^{n}-\omega_{N}^{n}\bigr) \bigr\Vert _{0}^{2} \\& \quad \le\sigma N^{2-2q}+ N^{-1}\sum _{i=0}^{n-1} \bigl\Vert \omega ^{i}- \omega_{N}^{i} \bigr\Vert _{0}^{2}, \quad n=1, 2, \ldots, M. \end{aligned}$$

By the discrete Gronwall inequality (Lemma 4) and from (51) we obtain

52 $$\begin{aligned}& \bigl\Vert \omega^{n}-\omega_{N}^{n} \bigr\Vert _{0}^{2}+\Delta t \bigl\Vert \nabla\bigl(\omega ^{n}-\omega_{N}^{n}\bigr) \bigr\Vert _{0}^{2} \\& \quad \le\sigma N^{2-2q}\exp\bigl(nN^{-1}\bigr) \\& \quad \le\sigma N^{1-2q},\quad n=1, 2, \ldots, M, 2\le q\le N+1. \end{aligned}$$

By (52) and Theorem 5 we obtain (35). Combining (52) with (47) and Theorem 5, we attain (36). This finishes the proof of Theorem 10. □

Because $\omega=\partial v/\partial x-\partial u/\partial y$ and $\omega_{N}^{n}=\partial v_{N}^{n}/\partial x-\partial u_{N}^{n}/\partial y$, we immediately attain the following result.

#### Theorem 11

*Under the same conditions as Theorems* 5 *and* 10, *the* 2*D non*-*stationary Stokes equations about vorticity–stream functions*, *Problem* 1, *has a unique set of fluid velocity CNFSE solutions* $(u_{N}^{n}, v_{N}^{n})$ *holding the following stability*:

53 $$ \bigl\Vert u_{N}^{n} \bigr\Vert _{0,\infty}+ \bigl\Vert v_{N}^{n} \bigr\Vert _{0,\infty}\le\sigma \Biggl[ \bigl\Vert u^{0} \bigr\Vert _{0,\infty}+ \bigl\Vert v^{0} \bigr\Vert _{0,\infty}+\Delta t N^{-1}\sum_{i=1}^{n}\bigl( \bigl\Vert g_{1}^{i} \bigr\Vert _{1,\infty}+ \bigl\Vert g_{2}^{i} \bigr\Vert _{1,\infty}\bigr) \Biggr], $$

*and the following convergence*:

54 $$ \bigl\Vert \partial_{y}\bigl(u(x,y,t_{n})-u_{N}^{n} \bigr) \bigr\Vert _{0}+ \bigl\Vert \partial_{x} \bigl(v(x,y,t_{n})- v_{N}^{n}\bigr) \bigr\Vert _{0}\le\sigma\bigl(\Delta t^{2}+N^{1-q}\bigr), $$

*where* $n=1,2, \ldots, M$ *and* $2\le q\le N+1$.

#### Remark 12

The error estimates in Theorem 11 attain optimal order even if *Θ* is the polygonal bounded domain and there is only $(u,v)\in H^{2}(0,T; H^{1}_{0}(\varTheta)\cap H^{2}(\varTheta))$. Especially, the system of equations (37) has sparse block-diagonal matrices with $4\times4$-blocks such that we enable to solve these equations numerically up to very large size of matrices by means of the chasing algorithm working with this kind of matrices by MATLAB software (see [10, 29]).

## Two numerical examples

In this section, we utilize three sets of numerical examples to verify the correctness of the theoretical results of the CNFSE format, i.e., Problem 8, for the 2D non-stationary Stokes equations about vorticity–stream functions. These numerical simulations are implemented by Matlab software on *Microsoft Surface Book*—Computer with Int Core i7 Processor and 16 GB RAM.

### The numerical example of square cavity flow

In this numerical example, we choose the computational field $\varTheta =(0, 1)\times(0, 1)$, $\mathit{Re}=10^{3}$, the side length $\Delta x=\Delta y=0.01$ of quadrilateral elements in $\Im_{N}$, i.e., $N=10^{4}$, the time step $\Delta t=0.0001$, the source vector function $(g_{1}(x,y,t),g_{2}(x,y,t))=(0,0)$, the initial velocity vector $(u^{0}(x,y),v^{0}(x,y))=(1, 0)$ on $0\le x\le1$ and $y=1$ but $(u^{0}(x,y),v^{0}(x,y))=(0, 0)$ on other part of *Θ̄*, the boundary value velocity vector $(\varphi_{u}(x,y,t),\varphi_{v}(x,y,t))=(1, 0)$ on $0\le x\le1$ and $y=1$ at $t=0$ but $(\varphi_{u}(x,y,t),\varphi_{v}(x,y,t))=(0, 0)$ on other sides of *∂Θ* and at other moments. Thus, we can conclude from $\|\partial_{y}(u_{N}^{n-1}-u_{N}^{n})\|_{0}+\|\partial _{x}(v_{N}^{n-1}- v_{N}^{n})\|_{0} =O(\Delta t^{2},N^{-2})$ that the theoretical errors for the CNFSE solutions are $O(10^{-8})$.

**Figure 1:**
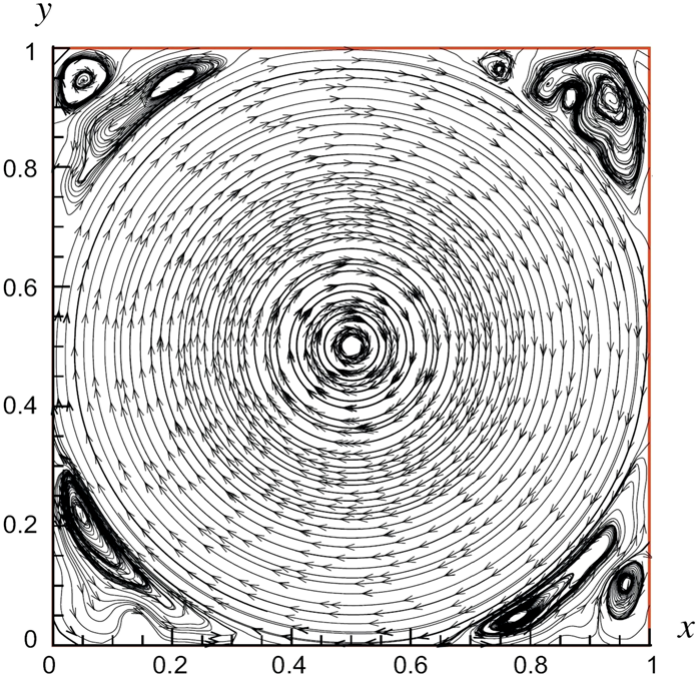
The CNFSE velocity solution when $t=3$

**Figure 2:**
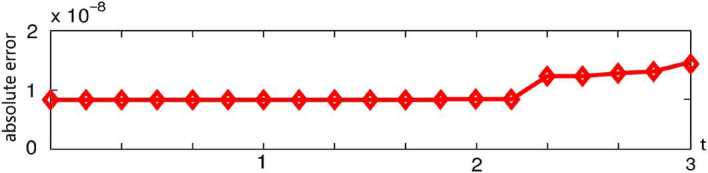
The absolute error when $0\le t\le3$ estimated by $\|\partial_{y}(u_{N}^{n-1}-u_{N}^{n})\|_{0}+\|\partial _{x}(v_{N}^{n-1}- v_{N}^{n})\|_{0}$ ($1\le n\le30\text{,}000$)

By the CNFSE model, i.e., Problem 2, we can compute out the CNFSE solution at $t =3$, depicted in Fig. 1. And the absolute error when $t=3$, estimated by $\|\partial_{y}(u_{N}^{n-1}-u_{N}^{n})\|_{0}+\| \partial_{x}(v_{N}^{n-1}- v_{N}^{n})\|_{0}$ ($1\le n\le30\text{,}000$), is depicted in Fig. 2, which are accorded with the theoretical conclusion, because both errors are no more than $O(10^{-8})$. This implies that the CNFSE model is efficient and feasible for solving the 2D non-stationary Stokes equations about vorticity–stream functions.

### The numerical example of channel flow with two identical rectangular protrusions

**Figure 3:**
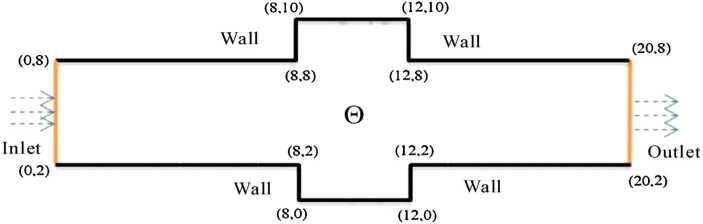
The computational domain and boundary conditions of flow

The computational domain *Θ* consists of a channel with a width of 6 and a total length of 20, with two identical rectangular protrusions at the bottom and at the top of the channel. The two rectangular protrusions both have a width of 2 and a length of 4 (see Fig. 3). A structured mesh with side length $\triangle x =\triangle y= 0.01$ is used. Except for the inflow from the left boundary with a velocity of $(u,v)=(0.1(y-2)(8-y),0)$ ($x=0$, $2\leq y\leq8$) and the outflow on the right boundary with velocity of $u(x,y,t)=u(20-1/N,y,t)$ ($20-1/N\leq x\leq20$, $2\leq y\leq8$, $0\leq t\leq T$), all of the initial and other boundary value conditions are taken as 0. The time-step increment is also taken as $\Delta t=0.0001$. In this case, the theoretical errors also attain $O(10^{-8})$.

**Figure 4:**
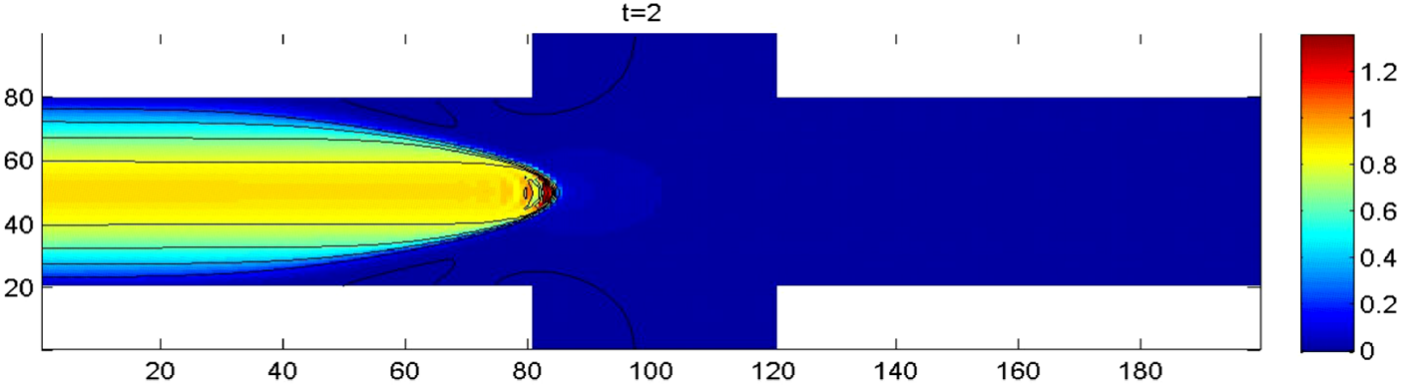
The CNFSE velocity solution when $t=2$

**Figure 5:**
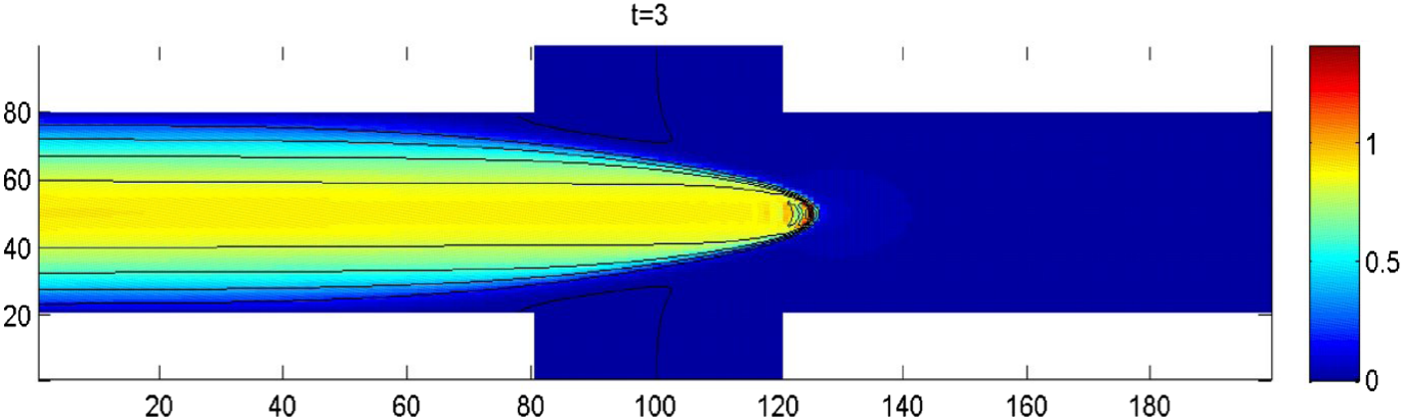
The CNFSE velocity solution when $t=3$

**Figure 6:**
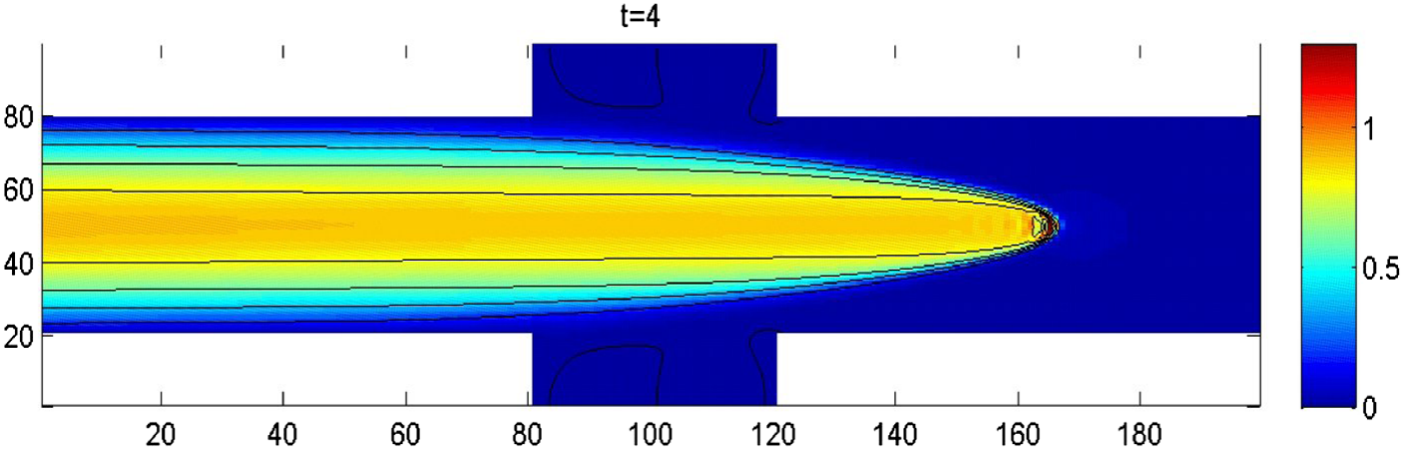
The CNFSE velocity solution when $t=4$

**Figure 7:**
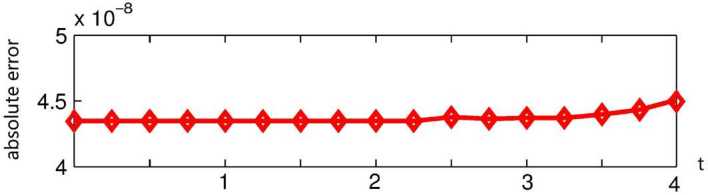
The absolute error when $0\le t\le4$ estimated by $\|\partial_{y}(u_{N}^{n-1}-u_{N}^{n})\|_{0}+\|\partial _{x}(v_{N}^{n-1}- v_{N}^{n})\|_{0}$ ($1\le n\le40\text{,}000$)

By the CNFSE model, i.e., Problem 2, we can compute out the CNFSE solutions at $t =2, 3, 4$, depicted in Figs. 4, 5, and 6, respectively. And the absolute error when $0 \le t\le4$, estimated by $\|\partial_{y}(u_{N}^{n-1}-u_{N}^{n})\|_{0}+\|\partial_{x}(v_{N}^{n-1}- v_{N}^{n})\|_{0}$ ($1 \le n\le40\text{,}000$), is depicted in Fig. 7, which are accorded with the theoretical conclusions, because both errors are no more than $O(10^{-8})$. This implies that the CNFSE model is valid and feasible for solving the 2D non-stationary Stokes equations about vorticity–stream functions.

### The numerical example with analytical solution

In this numerical example, we choose the computational field $\varTheta =(0, \pi)\times(0, \pi)$, the source function $f(x,y,t)=(1+2\mu) \exp(t) \sin x \sin y$, and $\omega^{0}= \sin x \sin y$ in (4). Thus, Eqs. (3) and (4) have a set of analytical solutions: $\psi=\omega/2=\frac{1}{2}\exp(t) \sin x \sin y$ and $\omega= \exp (t) \sin x \sin y$.

When $\mathit{Re}=10^{3}$, i.e., $\mu=10^{-3}$, we estimate the errors between the numerical solutions and the analytical solutions with different time steps and numbers of meshes at $t=1$ and 2, shown in Tables 1 and 2, respectively.

**Table 1:** The errors between the numerical solutions and the analytical solution at $t=1$

Δ*t* and *N*	$\|\nabla(\psi(x, y, t_{n})-\psi_{N}^{n})\|_{0}$	$\|\omega(x,y,t_{n})-\omega_{N}^{n}\|_{0}$
Δ*t* = 1/8 and *N* = 8	2.0951e−2	1.5036e−2
Δ*t* = 1/16 and *N* = 16	4.7351e−3	3.5732e−3
Δ*t* = 1/32 and *N* = 32	5.7826e−4	4.9875e−4
Δ*t* = 1/64 and *N* = 64	2.3564e−4	1.3161e−4

**Table 2:** The errors between the numerical solutions and the analytical solution at $t=2$

Δ*t* and *N*	$\|\nabla(\psi(x, y, t_{n})-\psi_{N}^{n})\|_{0}$	$\|\omega(x,y,t_{n})-\omega_{N}^{n}\|_{0}$
Δ*t* = 1/8 and *N* = 8	4.4732e−2	3.3764e−2
Δ*t* = 1/16 and *N* = 16	7.0124e−3	6.8274e−3
Δ*t* = 1/32 and *N* = 32	9.5675e−4	8.9906e−4
Δ*t* = 1/64 and *N* = 64	4.6703e−4	3.1033e−4

Tables 1 and 2 show that the numerically computing errors are accorded with the theoretical results in Theorem 10, i.e., both errors are second-order accuracy since $(1/8)^{2}=O(10^{-2})$, $(1/16)^{2}=O(10^{-3})$, $(1/32)^{2} = O(10^{-4})$, and $(1/64)^{2}=O(10^{-4})$. Due to the accumulation of round-off error, the numerically computing errors at $t=2$ are larger than those at $t=1$, which is reasonable. This further shows that the CNFSE model is efficient and feasible for finding the numerical solutions of the 2D non-stationary Stokes equations about vorticity–stream functions.

## Conclusions and discussion

In this work, we have established the time semi-discretized CN and CNFSE format for the 2D non-stationary Stokes equations about vorticity–stream functions and analyzed the existence, uniqueness, stability, and convergence of the time semi-discretized CN and CNFSE solutions, respectively. We have also used three sets of numerical examples to check the feasibility and effectiveness of the CNFSE format and to verity that the numerical computing consequences are accorded with the theoretical analysis ones. Moreover, it is shown that the CNFSE format is valid for solving the 2D non-stationary Stokes equations about vorticity–stream functions.

Although we here only research the CNFSE method for the 2D non-stationary Stokes equations about vorticity–stream functions, the CNFSE method can easily and effectively be used to solve for the non-stationary Stokes equations in three-dimensional space or more complex fluid dynamics equations, even be applied in the more complex real-world engineering problems. Therefore, our technique is promising as regards applications.
